# Community-Originated Research to Identify Access Gaps in Over-the-Counter Naloxone Availability in Connecticut Pharmacies

**DOI:** 10.1186/s12954-025-01268-y

**Published:** 2025-07-14

**Authors:** Katherine Hill, Peter Canning, Zoey Canning, Cameron Breen, Liz Evans, Mark Jenkins, Mark Nickel, Ken Plourd, Robert Heimer

**Affiliations:** 1 Department of Epidemiology of Microbial Diseases, Yale School of Public Health, New Haven, CT USA; 2 Emergency Medical Services, University of Connecticut John Dempsey Hospital, Farmington, CT USA; 3 Northwest Catholic High School, West Hartford, Connecticut, USA; 4 Liberation Programs, Bridgeport, CT USA; 5 CT Harm Reduction Alliance, Hartford, CT USA; 6 Cross Sector Consulting, LLP, Hamden, CT USA

**Keywords:** Naloxone, Narcan, Pharmacy

## Abstract

**Background:**

Naloxone, a life-saving medication that reverses opioid overdoses, was available in the United States only by prescription until March 2023, when the federal government approved nasal-spray formulations for over the counter sales to expand access. We assessed the availability of naloxone in a sample of pharmacies across the state of Connecticut.

**Methods:**

Between September 15 and November 24, 2024, trained community-based volunteers surveyed a convenience sample of pharmacies throughout the state, focusing on naloxone signage, availability, cost, and in-store location. Pharmacies were categorized into three groups: chain pharmacies, pharmacies within grocery stores, and independent pharmacies. Summary statistics for the full sample and the three subgroups were tabulated, and differences between groups were analyzed using Fisher’s exact tests.

**Results:**

A total of 162 pharmacies across all Connecticut counties were evaluated. While naloxone was available in most pharmacies, it was predominantly kept behind the pharmacy counter (n = 111, 73.5%) or the general checkout counter (n = 46, 30.5%). Fewer than 20% of pharmacies (n = 29) had naloxone easily accessible on an aisle shelf. Pricing was often high (≥ $60), particularly in independent pharmacies (n = 7, 22.6%; p < 0.001). Additionally, fewer than 20% of pharmacies (n = 31) displayed signage related to naloxone availability, and all signage was exclusively in English.

**Conclusions:**

Despite widespread availability, naloxone access was restricted by its in-store location, high cost, and inadequate signage. This highlights a notable discrepancy between naloxone availability and accessibility, suggesting a lag in the effective implementation of policy in intended settings.

**Supplementary Information:**

The online version contains supplementary material available at 10.1186/s12954-025-01268-y.

## Background

Naloxone binds to opioid receptors and, when administered during an opioid overdose, can reverse the overdose–potentially saving a life [1]. Between 2012 and 2022, fatal opioid-involved overdoses increased by almost fivefold in Connecticut (CT); in response measures were instituted to increase naloxone access [2]. In 2011, the state legislature passed one of the first ‘Good Samaritan Laws’ that protected against prosecution people who administered or prescribed naloxone [3]. Subsequent amendments strengthened the protections and expanded them to people who provided it. The state further expanded naloxone provision by creating standing orders allowing pharmacists to prescribe it after completing a training, supporting state financing of community distribution by first responders and harm reduction programs, offering voluntary dispensing to individuals upon release from incarceration, and providing Medicare and Medicaid coverage for prescriptions [4, 5]. In March 2023, the US Food and Drug Administration approved naloxone for over-the-counter (OTC) sales [6]. The purpose of these efforts was to maximize naloxone availability for community members to respond to overdoses and save lives.

Despite this policy effort, harm reduction programs around our state recognized that there are still parts of CT that were not reaching naloxone saturation–a condition by which providing additional naloxone to the community does not reduce overdose mortality [7]. CT’s harm reduction programs that distribute naloxone are centered around our major cities (e.g. Hartford, New Haven, Bridgeport). Even with an extensive harm reduction network in our state, a gap persists in naloxone distribution (e.g., harm reduction programs mostly serve urban centers, or those able to drive into them). As overdoses occur in every town, pharmacies can play a crucial role in increasing access to this live-saving medication. With the approval of OTC naloxone sales in pharmacies, we undertook this community-originated study to evaluate accessibility and availability of naloxone in pharmacies around the state. To date, few studies have evaluated the impact of such policy, though existing literature suggests gaps in OTC naloxone availability remain [8, 9].

## Methods

### Study Design

Our community-originated research design evaluated naloxone availability among a convenience sample of pharmacies in CT. Originally conceptualized as a volunteer student project that grew into a statewide initiative, community members were trained to enter pharmacies and systematically document naloxone availability. Our study team recruited 22 community-based volunteers–harm reductionists, healthcare providers, and college/graduate students–through the network of members of the CT Statewide Harm Reduction Partnership (SHaRP). To increase fidelity in surveyor responses while limiting the need for direct oversight surveyors received a written instruction guide (Additional file 1) and the structured survey tool (Additional file 2) that could be completed online using a tablet or smartphone. The Yale Human Research Protection Program determined that the activities of this project were not human subjects research (IRB Protocol ID: 2,000,039,492).

### Study Instrument and Sampling Scheme

Once trained, surveyors visited pharmacies with the online survey collecting the following pieces of information: (a) surveyor name, (b) pharmacy location and type, (c) naloxone signage, (d) naloxone availability, cost, and location in the store, and (e) conversations with pharmacists about naloxone training, prescriptions, insurance coverage, referrals, and the pharmacists’ use of any stigmatizing language. Surveyors had the ability to attach photos and include field notes based on their experiences. The pharmacies visited were based on a convenience sample; surveyors visited pharmacies near their places of residence or work.

### Data Analysis

Survey data were tabulated for the entire sample, and pharmacies were categorized into three groups based on previous research, expected patient experiences and business models in CT. Accordingly, we established the following three a priori groups: (1) chain pharmacies, as defined as large multi-location pharmacies (e.g., Walgreens, CVS, Rite Aid), (2) pharmacies in retail stores, defined as those within grocery stores or supermarkets (e.g., Stop & Shop, Walmart, Big Y), and (3) independently owned pharmacies, defined as smaller, often locally owned businesses such as single-store pharmacies, small chains, compounding pharmacies, or specialty pharmacies. Differences between these three groups were calculated through use of Fisher’s exact tests to account for small cell sizes. Analysis was conducted using R statistical software.

## Results

From September 15 to November 24, 2024, surveyors visited 162 pharmacies across all eight CT counties, covering 56 of 196 towns and 23 of 65 municipal health departments or regional health districts. Table 1 provides a summary of findings for all pharmacies and pharmacies stratified by pharmacy type.

**Table 1:** Naloxone availability in connecticut pharmacies, stratified by pharmacy type/location

Characteristic	Full Sample (n = 160) Freq (%)	Chain (n = 95) Freq (%)	Grocery Store (n = 34) Freq (%)	Independent (n = 31) Freq (%)	p-value
**Naloxone Availability within Store/Pharmacy**					0.442
Yes	151 (94.4)	91 (95.8)	32 (94.1)	28 (90.3)	
Naloxone Placement if Present (n = 151)*					-
Behind the pharmacy counter	111 (73.5)	59 (64.8)	24 (75.0)	28 (100.0)	< 0.001
Behind the general checkout counter	46 (30.5)	45 (49.5)	1 (3.1)	0 (0.0)	< 0.001
On an aisle shelf and easily accessible	29 (19.2)	21 (23.1)	8 (25.0)	0 (0.0)	0.005
On an aisle shelf with a theft deterrent	14 (9.3)	13 (14.3)	1 (3.1)	0 (0.0)	0.030
Naloxone Cost*					-
Less than $30	3 (1.9)	1 (1.1)	0 (0.0)	2 (6.5)	0.122
Between $30.00 and $39.99	22 (13.8)	21 (22.1)	1 (2.9)	0 (0.0)	< 0.001
Between $40.00 and $49.99	115 (71.9)	72 (75.8)	25 (73.5)	18 (58.1)	0.271
Between $50.00 and $59.99	7 (4.4)	2 (2.1)	3 (8.8)	2 (6.5)	0.111
$60 or more	12 (7.5)	1 (1.1)	4 (11.8)	7 (22.6)	< 0.001
Pricing not available	10 (6.3)	7 (7.4)	2 (5.9)	1 (3.2)	0.900
**Signage about Naloxone Availability**					0.086
Yes	31 (19.4)	24 (25.3)	4 (11.8)	3 (9.7)	
Signage Placement (n = 31) *					-
Pharmacy counter	18 (58.1)	13 (15.2)	3 (75.0)	2 (66.7)	0.829
Aisle	10 (32.3)	9 (37.5)	1 (25.0)	0 (0.0)	0.661
General checkout counter	4 (12.9)	4 (16.7)	0 (0.0)	0 (0.0)	1.000
Public-facing window / doorway entrance	2 (6.5)	2 (8.3)	0 (0.0)	0 (0.0)	1
Other	1 (3.2)	0 (0.0)	0 (0.0)	1 (33.3)	0.097
**Surveyor Spoke with Pharmacist About Accessing Naloxone**					0.758
Yes	149 (93.1)	87 (91.6)	32 (94.1)	30 (96.8)	
**Pharmacist Could Explain How to Access Naloxone at the Store or Why Naloxone Was Not Available (n = 149)**					1
Yes	141 (94.0)	82 (93.2)	30 (93.8)	29 (93.6)	
**Pharmacist Indicated Inability to Dispense Naloxone From Staff Not Being Trained or Authorized (n = 149)**					**0.022**
Yes	28 (18.8)	15 (15.5)	11 (34.4)	2 (6.7)	
**Pharmacist Discussed Insurance Coverage Options for Naloxone (n = 149)**					0.929
Yes	114 (76.5)	66 (75.9)	24 (75.0)	24 (80.0)	
**Pharmacist Offered to Write Prescription for Naloxone (n = 149)**					0.923
Yes	80 (53.7)	48 (55.2)	17 (53.1)	15 (50.0)	
**Pharmacist Offered Information / Referral to Naloxone In Community (n = 149)**					0.293
Yes	29 (19.5)	15 (15.5)	5 (15.6)	9 (30.0)	
**Pharmacist Was Sensitive with Use of Language (n = 149)**					0.959
Yes	120 (80.5)	69 (79.3)	26 (81.3)	25 (83.3)	

*Will not sum to 100%, as pharmacies could have signs/displays or naloxone in mulitple locations, or for various prices (e.g., generic vs. brand name)

### Naloxone Availability in Pharmacies

**Fig. 1:**
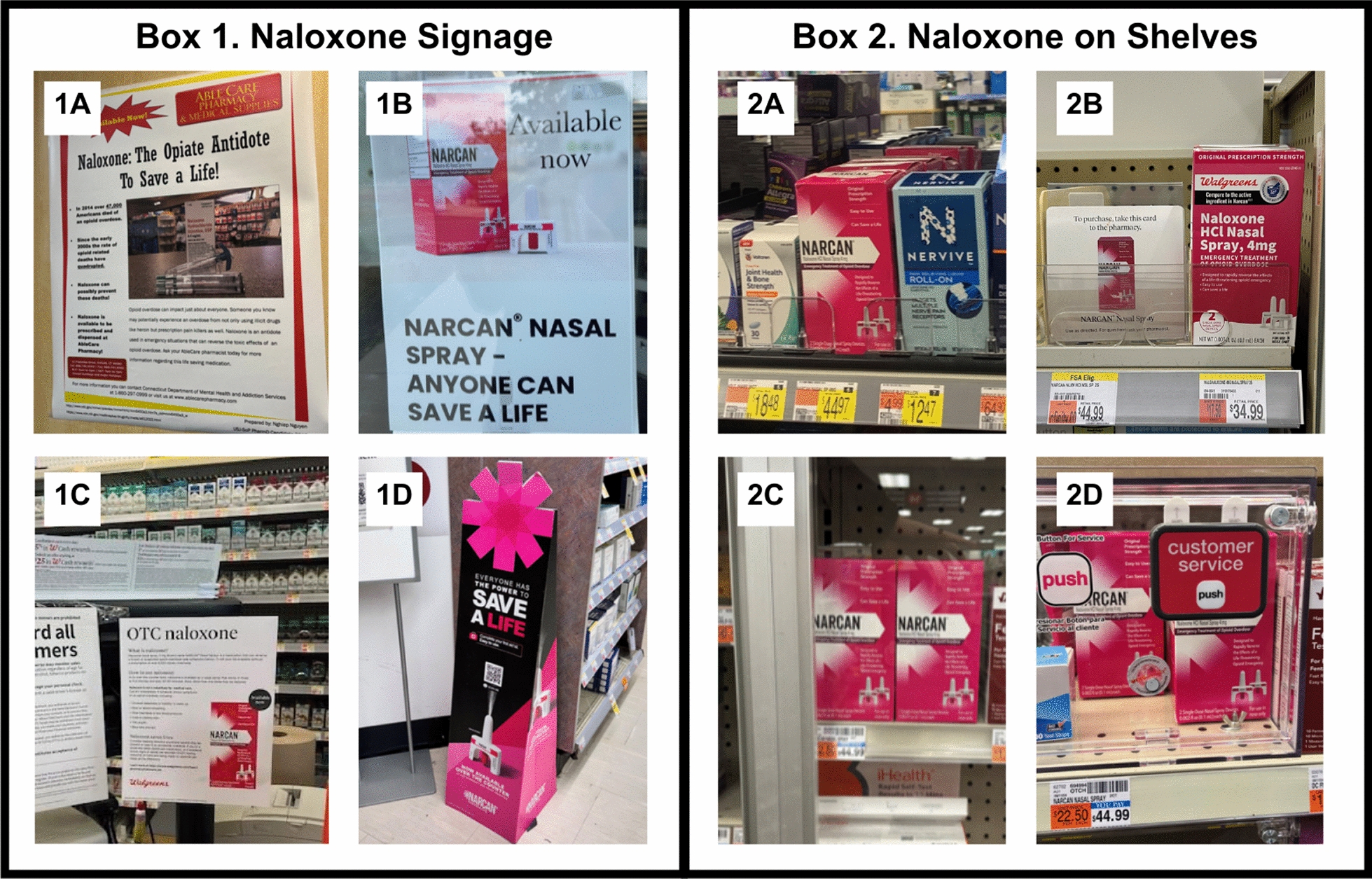
Naloxone signage and placement in pharmacies

Almost all the surveyed pharmacies had naloxone in stock (n = 151, 95.4%), with no statistically significant differences in availability by pharmacy type. However, among pharmacies with naloxone in stock, there was variation as to where customers could find the medication (see Fig. 1). Most sampled pharmacies kept naloxone behind the pharmacy counter (n = 111, 73.5%) or behind the general checkout counter (n = 46, 30.5%); conversely, less than a fifth of pharmacies (n = 29, 19.2%) had the medication easily available and accessible on an aisle shelf. Another 14 (9.3%) of pharmacies had naloxone on a shelf, though with a theft deterrent, such as a locked cabinet or with a redeemable coupon. All independent pharmacies stocking naloxone had it only behind the pharmacy counter while 64.8% (n = 59) and 75.0% (n = 24) of chain and grocery-store based pharmacies, respectively, had naloxone behind pharmacy counters. Chain pharmacies were most likely to have naloxone behind the general checkout counter (n = 45, 49.5%) and to have naloxone on a shelf but with theft deterrent packaging (n = 13, 14.3%). Only 21 chain pharmacies (23.1%) and eight pharmacies within supermarkets or grocery stores (25%) had naloxone easily accessible on an aisle shelf.

### Naloxone Cost in Pharmacies

Pharmacies were most likely to charge between $40 and $49.99 (n = 115, 71.9%) for naloxone, whether OTC or behind the counter. Field observations noted that name-brand Narcan™ was routinely sold for $44.99. A smaller proportion of pharmacies carried naloxone in a generic version and charged between $30 and $39.99 (n = 22, 13.8%). Chain pharmacies were the most likely to carry this version (n = 21, 22.1%, p < 0.001). Few pharmacies sold the product for under $30 (n = 3, 1.9%). Fourfold more sold it for ≥ $60 (n = 12, 7.5%), with independent pharmacies most likely to do so (n = 7, 22.6%, p < 0.001). It was noted in the field observations that one pharmacy was selling naloxone for as high as $180.

### Naloxone Signage in Pharmacies

Of the pharmacies surveyed, only about one-fifth of them had signs related to naloxone availability (n = 31, 19.4%); there were no statistically significant differences between the three types of pharmacies, though chain pharmacies had the highest prevalence of visible signage (n = 24, 25.3%) while independent pharmacies had the lowest prevalence (n = 3, 9.7%). All 31 visible signs and displays were only in English; most were placed on pharmacy counters (n = 18, 58.1%).

### Surveyor Conversations with Pharmacists

Regardless of pharmacy type, 95% of surveyors were able to speak to a pharmacist during 93.1% of surveys (n = 149). Most pharmacists were able to explain how to access naloxone in their store or explain why naloxone was not available (n = 141, 94.0%). Approximately three-quarters of pharmacists, regardless of pharmacy type, discussed insurance coverage options for naloxone (n = 114, 76.5%) and 80 (53.7%) offered to write a prescription for the medication.

We also observed differences in the ability of pharmacists to prescribe and dispense naloxone behind the pharmacy counter. Pharmacists at pharmacies within grocery stores or supermarkets were least likely to have received the necessary state-mandated training (11 of 32, 34.4% were not trained or authorized, p = 0.022). Very few pharmacists, across pharmacy types, offered any other information about or referred surveyors to other community resources to access naloxone (n = 29, 19.5%). However, most used sensitive, non-stigmatizing language when discussing naloxone (n = 120, 80.5%).

## Discussion

Though policy dictates naloxone should be available OTC across the US, our low-cost and efficient community-originated evaluation demonstrated a disconnect between availability and accessibility–implementation in the intended settings lags behind. Although widely available in CT, OTC naloxone is costly, primarily kept behind the counter, and accessibility differed by pharmacy type. Thus, initiatives to improve naloxone access may need to differ depending on the type of pharmacy. To start, chain pharmacies had the highest availability and accessibility of OTC naloxone (i.e., had naloxone in stock, in many locations), but had barriers such the presence of theft deterrents. While pharmacies within grocery stores and supermarkets frequently had naloxone behind the pharmacy counter available, more pharmacist training is needed to ensure staff are actually able to dispense this life-saving prescription. Lastly, independently owned (i.e., smaller, locally owned) pharmacies only had naloxone behind the pharmacy counter and this was often at a high cost. The results from our study thus provides an opportunity to inform professional development and strategic communication aimed at increasing the impact of policy changes and implement changes that improve naloxone access at pharmacies.

### Recommendations for Pharmacies

For stores that keep OTC naloxone behind the prescription counter, they should consider expanding shelf access to naloxone at multiple dosages/price points (e.g., Narcan™, RiVive™, etc.) outside of pharmacy hours. Pharmacies should make efforts to put up multilingual signage indicating naloxone availability and educate staff about its availability and store policies. To even further expand access to naloxone, pharmacies should ensure their pharmacists are trained and authorized to dispense this medication and do so without the use of stigmatizing language. Decisions to stock, advertise, and train employees will likely include stakeholders beyond pharmacists, such as store management or corporate teams.

### Recommendations for People Seeking Naloxone

Those hoping to access naloxone should note it may not be prominently displayed and might be available only behind the pharmacy counter. Thus, despite the recent federal approval of OTC naloxone, this life-saving medication may be available only when the pharmacy is open and if a customer knows to ask for it. Accordingly, we recommend people ask pharmacists for a prescription to lower costs, call ahead to confirm availability, and/or seek naloxone that is distributed for free through community-based harm reduction organizations.

### Study Limitations

Our study utilized a convenience sample of pharmacies in CT; thus, our findings may not be generalizable or transferable to other states. Additionally, results do not reflect official store policies and instead reflect experiences surveyors had at the time they visited the stores and the specific pharmacists with whom they spoke. Training of surveyors attempted to remove any systematic variation between volunteers, although variation in how surveyors assessed naloxone availability and spoke to pharmacists could exist; however, the use of a structured, closed-ended questionnaire should have mitigated errors in recording.

## Conclusions

While pharmacies have potential to serve as a great harm reduction tool, there is a need for widespread improvements to OTC naloxone accessibility and affordability in pharmacies. To increase accessibility, naloxone should be found at low cost in aisles without the need for prescription accompanied by signage indicating its location in the store. To increase affordability, pharmacists and healthcare providers should offer naloxone prescriptions to anyone filling a prescription for an opioid medication and anyone who requests a prescription.

## Supplementary Information

**Additional file 1**.

**Additional file 2**.

## Data Availability

No datasets were generated or analysed during the current study.
