# Supplementary material for: Community-Originated Research to Identify Access Gaps in Over-the-Counter Naloxone Availability in Connecticut Pharmacies
Source: Harm Reduct J. 2025 Jul 14;22:119. doi: 10.1186/s12954-025-01268-y (PMC12261670; doi:10.1186/s12954-025-01268-y)
Supplement: Supplementary file 1 — Additional file 1. [file 12954_2025_1268_MOESM1_ESM.docx]

**Quick Guide for Pharmacy Naloxone Survey**

Take picture of store prior to entering note time you enter. It may also help to screen shot the address as the survey monkey will ask for store address and time you visited. I find this helpful when visiting multiple stores to help keep them apart.

Look for signs for naloxone

Try to find naloxone on your own. Be sure to look behind front counter.

If you see an interesting sign or naloxone display, take a picture of it.

Go to pharmacy counter. If you didn’t see naloxone in store, confirm that they sell it.

Ask pharmacist if a person has insurance, will the pharmacist write them a prescription?

Ask if the pharmacy will sell 10 syringes to a person without a prescription.

Be polite, don’t engage in argument.

Write notes after leaving store.

Later, Fill out survey at this link:

Access to Naloxone in Pharmacies Inventory Tool Survey (surveymonkey.com)

https://www.surveymonkey.com/r/WSQSLXW

Survey will ask for health district. Attached is a list.

At end of survey, there is a box to add additional comments, please note if store was exceptionally helpful or not.

Thanks for helping with this.

| TOWN | local_health_department | COUNTY |
| --- | --- | --- |
| Andover | Eastern Highlands Health District | Tolland |
| Ansonia | Naugatuck Valley Health District | New Haven |
| Ashford | Eastern Highlands Health District | Windham |
| Avon | Farmington Valley Health District | Hartford |
| Barkhamsted | Farmington Valley Health District | Litchfield |
| Beacon Falls | Naugatuck Valley Health District | New Haven |
| Berlin | Central Connecticut Health District | Hartford |
| Bethany | Quinnipiack Valley Health District | New Haven |
| Bethel | Bethel Health Department | Fairfield |
| Bethlehem | Torrington Area Health District | Litchfield |
| Bloomfield | West Hartford-Bloomfield Health District | Hartford |
| Bolton | Eastern Highlands Health District | Tolland |
| Bozrah | Uncas Health District | New London |
| Branford | East Shore District Health Department | New Haven |
| Bridgeport | Bridgeport Health and Social Services | Fairfield |
| Bridgewater | Newtown Health District | Litchfield |
| Bristol | Bristol-Burlington Health District | Hartford |
| Brookfield | Brookfield Health Department | Fairfield |
| Brooklyn | Northeast District Department of Health | Windham |
| Burlington | Bristol-Burlington Health District | Hartford |
| Canaan | Torrington Area Health District | Litchfield |
| Canterbury | Northeast District Department of Health | Windham |
| Canton | Farmington Valley Health District | Hartford |
| Chaplin | Eastern Highlands Health District | Windham |
| Cheshire | Chesprocott Health District | New Haven |
| Chester | Connecticut River Area Health District | Middlesex |
| Clinton | Connecticut River Area Health District | Middlesex |
| Colchester | Chatham Health District | New London |
| Colebrook | Farmington Valley Health District | Litchfield |
| Columbia | Eastern Highlands Health District | Tolland |
| Cornwall | Torrington Area Health District | Litchfield |
| Coventry | Eastern Highlands Health District | Tolland |
| Cromwell | Cromwell Health Department | Middlesex |
| Danbury | Danbury Health and Human Services | Fairfield |
| Darien | Darien Health Department | Fairfield |
| Deep River | Connecticut River Area Health District | Middlesex |
| Derby | Naugatuck Valley Health District | New Haven |
| Durham | Connecticut River Area Health District | Middlesex |
| East Granby | Farmington Valley Health District | Hartford |
| East Haddam | Chatham Health District | Middlesex |
| East Hampton | Chatham Health District | Middlesex |
| East Hartford | East Hartford Health and Social Services | Hartford |
| East Haven | East Shore District Health Department | New Haven |
| East Lyme | Ledge Light Health District | New London |
| East Windsor | North Central District Health Department | Hartford |
| Eastford | Northeast District Department of Health | Windham |
| Easton | Aspetuck Health District | Fairfield |
| Ellington | North Central District Health Department | Tolland |
| Enfield | North Central District Health Department | Hartford |
| Essex | Essex Health Department | Middlesex |
| Fairfield | Fairfield Health Department | Fairfield |
| Farmington | Farmington Valley Health District | Hartford |
| Franklin | Uncas Health District | New London |
| Glastonbury | Glastonbury Health Department | Hartford |
| Goshen | Torrington Area Health District | Litchfield |
| Granby | Farmington Valley Health District | Hartford |
| Greenwich | Greenwich Health Department | Fairfield |
| Griswold | Uncas Health District | New London |
| Groton | Ledge Light Health District | New London |
| Guilford | Guilford Health Department | New Haven |
| Haddam | Connecticut River Area Health District | Middlesex |
| Hamden | Quinnipiack Valley Health District | New Haven |
| Hampton | Northeast District Department of Health | Windham |
| Hartford | Hartford Health & Human Services | Hartford |
| Hartland | Farmington Valley Health District | Hartford |
| Harwinton | Torrington Area Health District | Litchfield |
| Hebron | Chatham Health District | Tolland |
| Kent | Torrington Area Health District | Litchfield |
| Killingly | Northeast District Department of Health | Windham |
| Killingworth | Connecticut River Area Health District | Middlesex |
| Lebanon | Uncas Health District | New London |
| Ledyard | Ledge Light Health District | New London |
| Lisbon | Uncas Health District | New London |
| Litchfield | Torrington Area Health District | Litchfield |
| Lyme | Ledge Light Health District | New London |
| Madison | Madison Health Department | New Haven |
| Manchester | Manchester Health Department | Hartford |
| Mansfield | Eastern Highlands Health District | Tolland |
| Marlborough | Chatham Health District | Hartford |
| Meriden | Meriden Department of Health and Human Services | New Haven |
| Middlebury | Torrington Area Health District | New Haven |
| Middlefield | South Central Health District | Middlesex |
| Middletown | Middletown Health Department | Middlesex |
| Milford | Milford Health Department | New Haven |
| Monroe | Monroe Health Department | Fairfield |
| Montville | Uncas Health District | New London |
| Morris | Torrington Area Health District | Litchfield |
| Naugatuck | Naugatuck Valley Health District | New Haven |
| New Britain | New Britain Health Department | Hartford |
| New Canaan | New Canaan Health Department | Fairfield |
| New Fairfield | New Fairfield Health Department | Fairfield |
| New Hartford | Farmington Valley Health District | Litchfield |
| New Haven | New Haven Health Department | New Haven |
| New London | Ledge Light Health District | New London |
| New Milford | Housatonic Valley Health District | Litchfield |
| Newington | Central Connecticut Health District | Hartford |
| Newtown | Newtown Health District | Fairfield |
| Norfolk | Torrington Area Health District | Litchfield |
| North Branford | East Shore District Health Department | New Haven |
| North Canaan | Torrington Area Health District | Litchfield |
| North Haven | Quinnipiack Valley Health District | New Haven |
| North Stonington | Ledge Light Health District | New London |
| Norwalk | Norwalk Health Department | Fairfield |
| Norwich | Uncas Health District | New London |
| Old Lyme | Ledge Light Health District | New London |
| Old Saybrook | Connecticut River Area Health District | Middlesex |
| Orange | Orange Health Department | New Haven |
| Oxford | Housatonic Valley Health District | New Haven |
| Plainfield | Northeast District Department of Health | Windham |
| Plainville | South Central Health District | Hartford |
| Plymouth | Torrington Area Health District | Litchfield |
| Pomfret | Northeast District Department of Health | Windham |
| Portland | Chatham Health District | Middlesex |
| Preston | Uncas Health District | New London |
| Prospect | Chesprocott Health District | New Haven |
| Putnam | Northeast District Department of Health | Windham |
| Redding | Redding Health Department | Fairfield |
| Ridgefield | Ridgefield Health Department | Fairfield |
| Rocky Hill | Central Connecticut Health District | Hartford |
| Roxbury | Newtown Health District | Litchfield |
| Salem | Uncas Health District | New London |
| Salisbury | Torrington Area Health District | Litchfield |
| Scotland | Eastern Highlands Health District | Windham |
| Seymour | Naugatuck Valley Health District | New Haven |
| Sharon | Housatonic Valley Health District | Litchfield |
| Shelton | Naugatuck Valley Health District | Fairfield |
| Sherman | Sherman Health Department | Fairfield |
| Simsbury | Farmington Valley Health District | Hartford |
| Somers | Somers Health Department | Tolland |
| South Windsor | South Windsor Health Department | Hartford |
| Southbury | Housatonic Valley Health District | New Haven |
| Southington | South Central Health District | Hartford |
| Sprague | Uncas Health District | New London |
| Stafford | North Central District Health Department | Tolland |
| Stamford | Stamford Health Department | Fairfield |
| Sterling | Northeast District Department of Health | Windham |
| Stonington | Ledge Light Health District | New London |
| Stratford | Stratford Health Department | Fairfield |
| Suffield | North Central District Health Department | Hartford |
| Thomaston | Torrington Area Health District | Litchfield |
| Thompson | Northeast District Department of Health | Windham |
| Tolland | Eastern Highlands Health District | Tolland |
| Torrington | Torrington Area Health District | Litchfield |
| Trumbull | Trumbull Health Department | Fairfield |
| Union | Northeast District Department of Health | Tolland |
| Vernon | North Central District Health Department | Tolland |
| Voluntown | Uncas Health District | New London |
| Wallingford | Wallingford Health Department | New Haven |
| Warren | Torrington Area Health District | Litchfield |
| Washington | Housatonic Valley Health District | Litchfield |
| Waterbury | Waterbury Health Department | New Haven |
| Waterford | Ledge Light Health District | New London |
| Watertown | Torrington Area Health District | Litchfield |
| West Hartford | West Hartford-Bloomfield Health District | Hartford |
| West Haven | West Haven Health Department | New Haven |
| Westbrook | Westbrook Health Department | Middlesex |
| Weston | Aspetuck Health District | Fairfield |
| Westport | Aspetuck Health District | Fairfield |
| Wethersfield | Central Connecticut Health District | Hartford |
| Willington | Eastern Highlands Health District | Tolland |
| Wilton | Wilton Health Department | Fairfield |
| Winchester | Torrington Area Health District | Litchfield |
| Windham | North Central District Health Department | Windham |
| Windsor | Windsor Health Department | Hartford |
| Windsor Locks | North Central District Health Department | Hartford |
| Wolcott | Chesprocott Health District | New Haven |
| Woodbridge | Quinnipiack Valley Health District | New Haven |
| Woodbury | Housatonic Valley Health District | Litchfield |
