# Supplementary material for: Community-Originated Research to Identify Access Gaps in Over-the-Counter Naloxone Availability in Connecticut Pharmacies
Source: Harm Reduct J. 2025 Jul 14;22:119. doi: 10.1186/s12954-025-01268-y (PMC12261670; doi:10.1186/s12954-025-01268-y)
Supplement: Supplementary file 2 — Additional file 2. [file 12954_2025_1268_MOESM2_ESM.pdf]

1. Please enter your first and last name.

First name

Last name

2. Please enter the date of your visit.

Visit Date

Date

MM/DD/YYYY

3. Please enter the time of your visit.

Time of Visit

Time AM/PM

hh mm -

4. Which Pharmacy did you visit?

- CVS
- Walgreens
- Stop & Shop
- Costco
- Beacon
- Arrow
- Other (please specify)

5. Please enter the street address and town of the pharmacy.

Street Address

Town

6. Please select the health district in which the pharmacy was located.

SIGNAGE

7. Did you see any signs or displays about naloxone availability?

- ◯ Yes
- ◯ No

8. Where was the information displayed? (check all that apply)

- ☐ Public-facing window or doorway entrance
- ☐ Aisle sign or display
- ☐ Pharmacy counter
- ☐ Other (please specify)

9. What language did the sign use? (check all that apply)

- ☐ English
- ☐ Spanish
- ☐ Other (please specify)

10. Did you take a picture of the signage?

- ◯ Yes
- ◯ No

11. Please upload your picture of the signage.

Choose File Choose File No file chosen

ACCESSIBILITY TO SYRINGES

12. Does the pharmacy have syringes in stock?

- ◯ Yes
- ◯ No

13. Does the pharmacy sell the syringes to consumers without a prescription?

- ◯ Yes
- ◯ No

ACCESSIBILITY TO NALOXONE

14. Where is the naloxone located? (check all that apply)

- [ ] On an aisle shelf – easily accessible
- [ ] On an aisle shelf with a theft deterrent such as a lock cabinet or a redeemable coupon
- [ ] Behind the general checkout counter
- [ ] Behind the pharmacy counter
- [ ] Other (please specify)

15. Did you take a picture of the shelf accessibility?

- ○ Yes
- ○ No

16. Please upload your picture of the shelf accessibility.

Choose File Choose File No file chosen

COST

17. How much did the naloxone cost?

- ○ Less than $29.99
- ○ Between $30.00 and $39.99
- ○ Between $40.00 and $49.99
- ○ Between $50.00 and $59.99
- ○ Over $60
- ○ Other (please specify)

KNOWLEDGE BY PHARMACIST

18. Did you ask to speak with a **pharmacist** about accessing naloxone?

- ○ Yes
- ○ No

19. Was the pharmacist helpful in explaining how to access naloxone at the store or why naloxone was not available at the store?

- ◯ Yes
- ◯ No

20. Did the pharmacist indicate that the store was not able to dispense naloxone because the pharmacist was not trained or authorized to dispense naloxone?

- ◯ Yes
- ◯ No

21. Did the pharmacist discuss options for using insurance coverage to access naloxone?

- ◯ Yes
- ◯ No

22. Did the pharmacist offer to write a prescription for naloxone?

- ◯ Yes
- ◯ No

23. Did the pharmacist offer you any other information or refer you to other community resources to access naloxone?

- ◯ Yes
- ◯ No

24. Did you perceive that the pharmacist was sensitive with use of language (i.e., stigma free)?

- ◯ Yes
- ◯ No

KNOWLEDGE BY CUSTOMER SERVICE REPRESENTATIVE

25. Did you ask a **customer service representative** working in the store about accessing naloxone?

- ◯ Yes
- ◯ No

26. Was the customer service representative helpful in explaining how to access naloxone at the store or why naloxone was not available at the store?

- ◯ Yes
- ◯ No

27. Did you perceive that the customer service representative was sensitive with the use of language (i.e., stigma free)?

- ◯ Yes
- ◯ No

OTHER OBSERVATIONS

28. Please enter any other notes and/or observations from your visit.
